# No evidence for stochastic resonance effects on standing balance when applying noisy galvanic vestibular stimulation in young healthy adults

**DOI:** 10.1038/s41598-021-91808-w

**Published:** 2021-06-10

**Authors:** L. Assländer, L. S. Giboin, M. Gruber, R. Schniepp, M. Wuehr

**Affiliations:** 1 Human Performance Research Centre, University of Konstanz, Konstanz, Germany; 2 German Center for Vertigo and Balance Disorders (DSGZ), Ludwig-Maximilians-University, Munich, Germany; 3 Department of Neurology, Ludwig-Maximilians-University, Munich, Germany

**Keywords:** Neuroscience, Motor control, Neurophysiology

## Abstract

Noisy galvanic vestibular stimulation (nGVS) at imperceptible levels has been shown to reduce body sway. This reduction was commonly attributed to the mechanism of stochastic resonance (SR). However, it has never been explicitly tested whether nGVS-induced effects on body sway consistently follow a SR-like bell-shaped performance curve with maximal reductions in a particular range of noise intensities. To test this, body sway in 21 young healthy participants was measured during varying nGVS amplitudes while standing with eyes closed in 3 conditions (quiet stance, sway referencing, sinusoidal platform tilts). Presence of SR-like response dynamics in each trial was assessed (1) by a goodness-of-fit analysis using an established SR-curve model and (2) by ratings from 3 human experts. In accordance to theory, we found reductions of body sway at one nGVS amplitude in most trials (75–95%). However, only few trials exhibited SR-like bell-shaped performance curves with increasing noise amplitudes (10–33%). Instead, body sway measures rather fluctuated randomly across nGVS amplitudes. This implies that, at least in young healthy adults, nGVS effects on body sway are incompatible with SR. Thus, previously reported reductions of body sway at particular nGVS intensities more likely result from inherent variations of the performance metric or by other yet unknown mechanisms.

## Introduction

Stochastic resonance (SR) is a phenomenon in non-linear systems, where the system's response to weak, sub-threshold signals becomes enhanced in the presence of a weak non-zero amount of noise^1,2^. In general, SR effects critically depend on the noise amplitude. Very small noise levels show hardly any effects on the signal transmission at the non-linearity (i.e., the threshold). When increasing the noise amplitude, the superposition of noise and encoded signal will reach a point where, in systems susceptible to SR, the signal transmission around the threshold gets improved. Larger noise amplitudes will, however, increasingly add variability to the signal and ultimately disturb signal transmission. Accordingly, the characteristic signature for the presence of SR is a noise-induced modulation of the system's output that follows a pseudo-bell-shaped curve, which peaks at some particular level of noise that optimally facilitates signal transfer within the system^3,4^.

SR-like effects have been demonstrated in animal models at the afferent receptor level for a variety of sensory systems, such as the somatosensory, auditory, and visual system^5–9^. Similar effects have been later verified in humans in terms of improvements at the perceptual level as well as in related sensorimotor function^3,10–14^. More recently, analogous effects have been observed in the vestibular system (for a review see^15,16^). To induce SR-like effects in the vestibular system, most studies used galvanic vestibular stimulation (GVS). GVS is a non-invasive technique that allows researchers to electrically modulate the neuronal activity in the peripheral vestibular endorgans^17^. By applying a noisy form of GVS (nGVS) to healthy young individuals, Galvan-Garza and colleagues could demonstrate that vestibular motion perception thresholds can get effectively reduced in the presence of a low-intensity stochastic vestibular stimulation^18^. Accordingly, they observed that in about 75% of participants nGVS-induced modulations of thresholds (assessed during a direction recognition task) followed a bell-shaped performance curve with increasing noise amplitude. In agreement with the aforementioned theoretical framework of SR the authors observed a minimum of the threshold at an intermediate nGVS intensity of about 200 to 500 μA peak amplitude. These observations were later confirmed and extended in subsequent studies^19–21^.

To date, vestibular SR effects have been most extensively studied in the context of human balance control. Reductions in postural sway when applying nGVS have been reported for young and older healthy individuals^22,23^ as well as in patients with peripheral vestibular hypofunction^24^ or central neurodegenerative diseases^25^. These reports have been complemented by studies that specifically analyzed the effects of different stimulus characteristics^26,27^, varying conditions of standing^28–30^ as well as immediate and after effects of stimulation^31–34^ on spontaneous sway patterns. In light of these studies, vestibular SR has been proposed to have immediate clinical implications in terms of a potential treatment option for postural imbalance in the context of a peripheral and/or central vestibular dysfunction^15^.

One common shortcoming of previous reports on nGVS effects on balance is, however, that they didn't provide sufficient evidence that the observed beneficial effects of nGVS on balance control are actually compatible with the presence of vestibular SR-like behavior. Accordingly, none of these studies demonstrated that nGVS-induced modulations of body sway consistently follows a bell-shaped curve with increasing nGVS amplitude. Rather, some studies examined the effects of only one fixed nGVS amplitude versus sham stimulation^22,23,25,27–30,33,34^. Others studied the effects of different nGVS amplitudes but only reported performance modulations at the amplitude at which individuals showed optimally improved balance^24,26,31,32^. This approach, however, is statistically problematic since a seeming reduction in sway across several repeated assessments would be likely expected simply due to inherent test–retest variations in the metric. Thus, both approaches fail to provide convincing evidence that reported reductions in postural sway are linked to the mechanism of SR.

The aim of this study was therefore to examine whether previously reported nGVS-induced reductions in postural sway are consistent with and may be explained by the exhibition of SR. We hypothesized that, in analogy to previously reported effects of nGVS on vestibular perception^18^, nGVS-induced reductions of body sway would follow a bell-shaped performance curve with increasing stimulation intensity consistent with the presence of SR. To this end, we systematically analyzed modulations of body sway in dependence of varying intensities of nGVS in young healthy individuals. nGVS-induced changes in body sway were studied in eyes closed stance during three surface conditions: fixed, sway referenced, and sinusoidally tilting. The fixed surface condition was used in most studies discussed above. We further examined sway referencing of the support surface during which the postural control mechanism almost exclusively depends on vestibular inputs and should be more sensitive to nGVS^35^. Lastly, sway responses to a sinusoidal tilt stimulus were examined since they reveal changes in the postural control dynamics independent from changes in internal noise and resulting sway variability^36^. We applied different quantitative and qualitative criteria on a single subject level to determine whether nGVS-induced modulations in body sway were consistent with the presence of SR or rather follow other response dynamics.

## Materials and methods

### Participants

Twenty-one healthy young subjects (age: 24.0 ± 4.2 years; height: 175 ± 10 cm; weight: 69.5 ± 12.4 kg, 11 females), the majority recruited from students of the university, participated in this study. None of the participants reported any auditory, vestibular, cardio-vascular, or orthopedic disorders. All participants gave their written informed consent prior to the experiments. The Ethics Committee of the Ludwig-Maximilians-University approved the study protocol, which was conducted in conformity with the Declaration of Helsinki.

### Experimental setup

**Figure 1:**
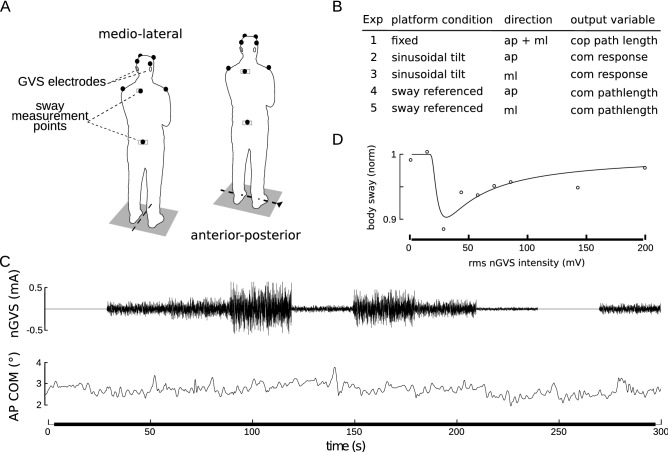
Summary of the experimental setup. (**A**) the two stance positions on the tilt platform and marker placements. (**B**) list of experimental conditions. (**C**) example of the nGVS signal and anterior–posterior COM body sway during quiet stance. The order of nGVS amplitudes was randomized for each subject. (**D**) exemplary modulation of body sway across the tested nGVS intensities (simulated data) that follows a SR-like pseudo-bell-shaped curve with corresponding curve fit^4,18^.

During experiments, subjects stood either on two force plates (AMTI, Watertown, USA) or on a custom-built motorized tilt platform. The tilt platform was 60 × 60 cm in diameter, servo-controlled and had the rotation axis approximately at the height of the ankle joints (8.8 cm above the support)^37^. Body sway was measured using a camera-based motion capture system (Vicon, Cambridge, UK). Reflective markers were attached to the sacrum, between the shoulder blades, and—not used for analyses—at head and shoulders (Fig. 1A). Stimuli were generated using Matlab scripts and a Simulink model (The Mathworks, Natick, USA) running on a real-time Target PC (Speedgoat, Switzerland). Analog (stimuli, force plates) and camera data were recorded on a PC running Nexus software (Vicon, Cambridge, UK) at 2000 Hz (down-sampled to 100 Hz for the analysis) and 100 Hz sampling rate, respectively. We applied rubber-electrodes (approx. 10 cm^2^ area) with electrode gel to the mastoid process behind each ear and fixed them with a head band. The electrodes were connected to an isolated bipolar current stimulator (Digitimer, Hertfordshire, UK) with the noise stimulus sequence as input.

### Galvanic stimulus sequences and platform conditions

Zero mean white noise sequences with a variance of 1 mA of 30 s or 60 s duration were generated using the ‘wng’ function (Matlab, ‘Communications Toolbox’) and filtered using a second order Butterworth band-pass filter with 0.02 and 10 Hz cutoff frequencies. In accordance to previous studies, this stimulation bandwidth was chosen to cover most of the dynamic range of motion signals encoded by the peripheral vestibular system as well as the range of head motion signals that typically occur during upright standing^24,31,32^. The noise sequence was applied 9 consecutive times, with different scaling factors (0; 0.1; 0.2; 0.3; 0.4; 0.5; 0.6; 1.0; 1.4), which resulted in signal root-mean-square (RMS) values of 0, 14, 28, 43, 57, 71, 85, 142, and 199 μA, which approximately correspond to peak-amplitude values of 0, 0.05, 0.10, 0.15, 0.20, 0.25, 0.30, 0.50, 0.70 mA. Each platform condition started with a 30 s period without galvanic stimulation, followed by the 9 nGVS amplitudes (including another 0-mA condition) in randomized order. Each noise amplitude was applied for 30 s during the fixed surface and sway referencing conditions and for 60 s during the sine condition (to ensure enough tilt cycles for averaging the response to the sinusoidal stimulus). The sine conditions were split up into two trials with 4 and 5 different 60-s long nGVS amplitudes. A new noise sequence and a new random order of stimulus amplitudes was generated for each subject.

The nine nGVS amplitudes were tested with eyes closed in three different platform conditions: fixed surface, sway referenced surface, and sinusoidal tilting surface (Fig. 1B). Sway referencing is a condition, in which the platform is commanded to follow the sway of the subject, such that orientation of the subjects’ legs is not changing with respect to the platform. Sway referencing was achieved by measuring the movement of the hip via a sway-rod attached to a potentiometer, that was guided by a hook attached to the subjects' hip. Trigonometric calculations were used to calculate the command signal for the platform. As a result, proprioceptive information on body orientation with respect to the platform is almost decoupled from body orientation in space. The sine had a frequency of 0.5 Hz and 0.5° amplitude and was started with a raised cosine to avoid platform jerks. Sway reference and sinusoidal tilt conditions were applied in separate trials for anterior–posterior (AP) and medio-lateral (ML) directions, while both directions were simultaneously recorded in quiet stance.

### Procedures

After providing written informed consent, subjects’ anthropometric measures were taken. Thereafter, we attached the reflective markers, hooks for sway referencing and nGVS electrodes after skin preparation with abrasive gel and disinfection. Subjects were asked to perform a 120-s long calibration routine in AP and ML direction (see below). The different stance conditions were presented in randomized order with short breaks of 1–2 min in between and longer breaks if requested by the subjects. Subjects were instructed to close the eyes and ‘stand upright and comfortable’ and were listening to non-rhythmic audio-books via noise-canceling head-phones to avoid auditory orientation cues and distract from the balancing task.

### Data analysis

Center of pressure (COP) was calculated for quiet stance trials as output measure. Center of mass was calculated for sway referencing and sine trials as output measure, since the calculation of the COP was technically not feasible during moving platform conditions.

Center of mass (COM) was obtained from hip and shoulder movements using a calibration routine^38,39^. During the calibration routine, subjects performed slow movements in the ankle and hip joints. Recorded COP trajectories were used as a projection of the COM position in these quasi-static trials. Assuming two-segment mechanics consisting of leg and head-arms-trunk segments, a regression between hip and ankle kinematics (x_COP_ ≈ x_COM_ = Off + A · x_hip_ + B · x_sho_) provides calibration factors (Off, A, B), which can be used to obtain x_COM_ sway in conditions where x_COM_ ≠ x_COP_. Finally, COM angle is calculated from x_COM_ and COM height, as obtained from subject anthropometrics^40^. The calibration routine and calculations were done for AP and ML directions independently.

To confirm the well documented effect of binaural bipolar GVS evoking a postural response in ML direction when the head is facing forward^41,42^ cumulant density functions between quiet stance COP and nGVS stimulus sequences with corresponding 95% confidence bounds were calculated for the first 28.16 s of each nGVS amplitude and across all subjects (using neurospec toolbox version 2.11^43,44^). For COP in quiet stance and COM in sway referenced platform conditions, path-length was calculated for each nGVS condition and subject using p = 1/T · ∑_i_ |x_i+1_-x_i_|, where T is the trial duration and x_i_ are the individual samples. For the sine condition, body sway amplitude in response to the stimulus was obtained from the sway amplitude spectrum at the stimulus frequency calculated using a scaled Fast Fourier Transform. Data for individual subjects were normalized to body sway measures obtained during the 0-µA nGVS condition and mean +/− standard deviation across subjects were calculated.

For each trial, we initially determined whether body sway measures improved for at least one particular nGVS level compared to baseline condition (i.e., 0 µA nGVS). Since there is no established mathematical definition of what suffice for the exhibition of SR-like dynamics in response to varying levels of nGVS, we tested two alternative SR criteria, both on the results of single individuals as well as on the group outcomes. For the first criterion, we performed a goodness-of-fit analysis using an established SR-curve fit on the relationship between nGVS levels and normalized body sway measures as proposed in Galvan-Garza et al.^18^. The SR function fits a bell-shaped curve (see Fig. 1D) on the data based on an equation that was initially developed to describe the general phenomenon of SR^4^. A limitation of this model is, that it does not cover potential increases of body sway measures above baseline level at higher stimulation amplitudes. The SR function fit is given by:

$$ A = B + A_{0} *\frac{\lambda }{{q_{2} }}*\frac{1}{{\sqrt {4r + {\Omega }^{2} } }} $$

$$ {\text{with}}\quad r = \frac{1}{\sqrt 2 *\pi }*\lambda *e^{{ - \frac{{\lambda^{2} }}{{2*q_{2} }}}} $$

$$ {\text{and}}\quad q_{2} = q + dq $$

where A_0_, Ω, and λ determine the depth of the bell, B the y-axis offset, q the x-axis (nGVS amplitude), and dq the x-axis offset. All five parameters were fitted to experimental data using a global optimization approach using the function ‘fmincon’ and ‘GlobalSearch’ option from the Matlab ‘Global Optimization Toolbox’. Subsequently, we tested for each trial whether the SR curve fit can better explain the experimental data compared to a simple linear fit. The comparison was performed using F-test statistics to account for the trade-off between improved residuals and additional free parameters in each fit with *F*_3,4_ = 6.59 for *p* < 0.05. The second criterion was more subjective. In accordance to a previous study, three authors (who were all familiar with the expected SR-like curve shape) were asked to judge for each single subject and condition—without any additional predefined criteria—whether SR-like behavior is present based on visual inspection of the data and the corresponding linear and SR curve fits^18^. This criterion required that at least two of the raters identified the presence of SR-like dynamics in a trial.

## Results

During all examined stance conditions (quiet stance, sway-referencing, sinusoidal tilt), participants were able to maintain stable balance, except in four single trials (two due to circulation problems and two due to repeated loss of balance during sway referencing) that were excluded from further analysis. During quiet stance with eyes closed, subjects showed slight forward body lean and typical body sway amplitudes (exemplary COM body sway of one participant is presented in Fig. 1C). Body sway was 3–4 times larger in the sway referenced condition (not shown) as compared to quiet stance on a fixed surface. During the sinusoidal tilt condition, platform movement evoked sway responses that were visible in the time domain for most subjects. However, they were superimposed by considerable random sway.

**Figure 2:**
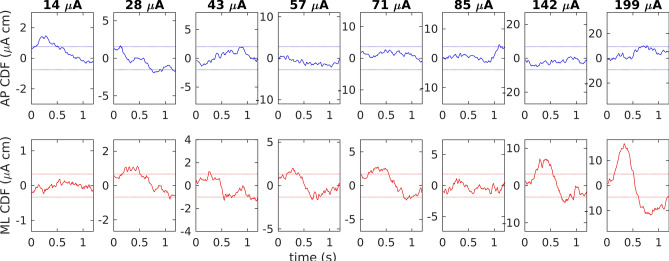
Cumulant density functions between COP and nGVS sequences for different stimulus amplitudes during quiet stance for anterior–posterior (blue, top row) and medio-lateral (red, bottom row) directions. Plots show the analysis results comprising all subjects. Dashed lines show 95% confidence bounds; plots are scaled with respect to the confidence bounds.

In a first step of analysis, we examined whether nGVS above a certain intensity level (range of applied intensities: 0–199 µA signal RMS) would evoke a postural response in analogy to previously reported effects of GVS on ML postural sway^41^. Visual inspection of corresponding nGVS and body sway traces did not exhibit obvious patterns of covariance (Fig. 1C). Cumulant density estimates (Fig. 2), however, revealed significant coupling between nGVS and body sway at the two highest applied nGVS intensities (i.e., 142 and 199 µA signal RMS). This effect was only observed for body sway in ML but not in AP direction. Cumulant density estimates further exhibited a characteristic biphasic pattern with short and medium latency responses at around 350 and 700 ms in agreement with previous reports^45,46^. Hence, this analysis confirmed that the range of applied nGVS stimuli encompassed both suprathreshold nGVS intensities that have a direct modulatory effect on body sway as well as subthreshold nGVS intensities that could potentially induce alterations in body sway via vestibular SR.

**Figure 3:**
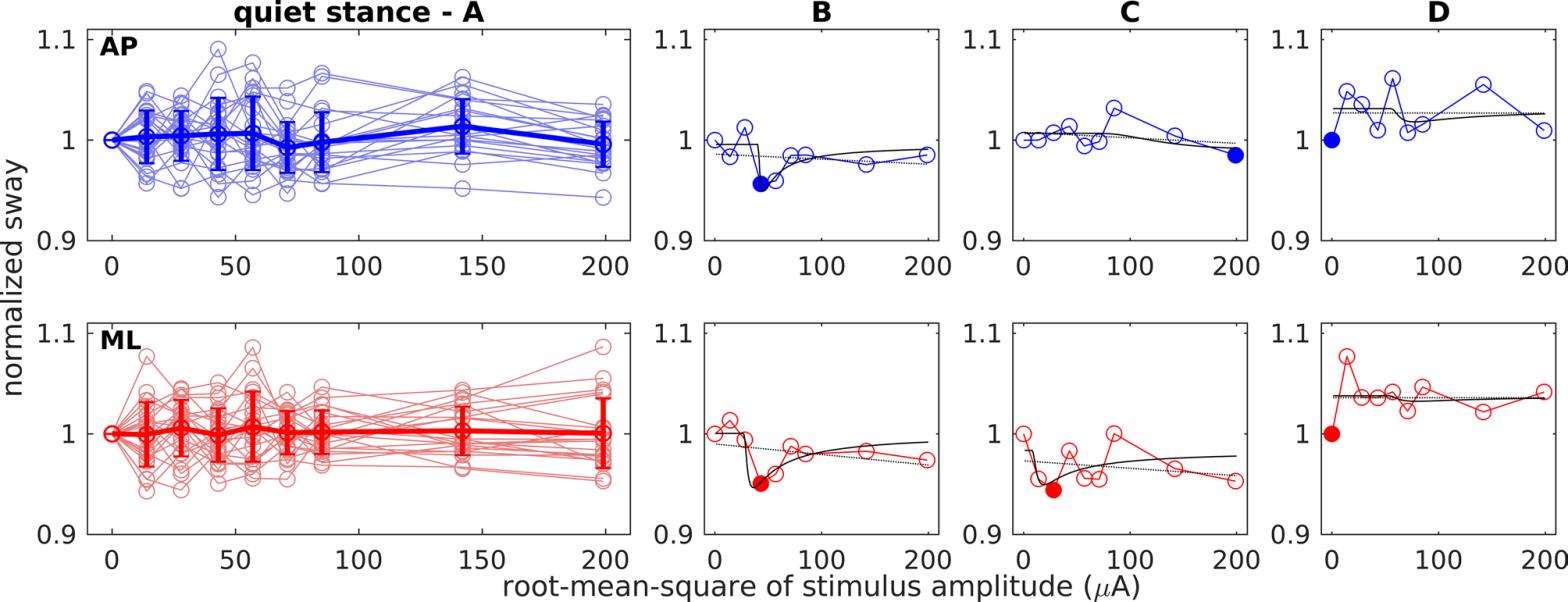
Normalized body sway path during quiet stance at varying nGVS intensities. Top row (AP, blue) shows anterior–posterior sway. Bottom row (ML, red) shows medio-lateral sway. (**A**) Responses of all subjects (light circles and lines) with group mean and standard deviation (thick line). (**B–D**) Three exemplary individual responses including the linear (grey) and the SR curve fit (black). Filled circles indicate minimum values. (**B**) Performance curve showing a SR-like bell-shaped with optimal improvement at an intermediate nGVS intensity. (**C**) Performance curve showing only optimal improvement at a particular nGVS intensity. (**D**) Performance curve showing none of the two.

**Figure 4:**
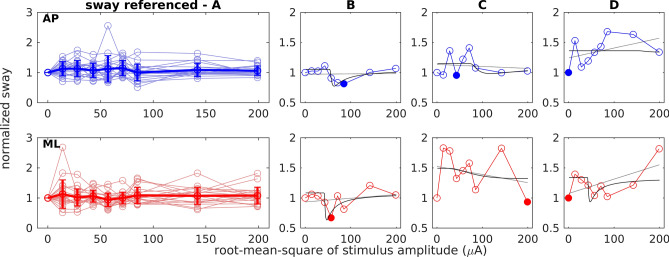
Normalized body sway path for standing with sway-referencing at varying nGVS intensities. Top row (AP, blue) shows anterior–posterior sway. Bottom row (ML, red) shows medio-lateral sway. (**A**) Responses of all subjects (light circles and lines) with group mean and standard deviation (thick line). (**B–D**) Three exemplary individual responses including the linear (grey) and the SR curve fit (black). Filled circles indicate minimum values. (**B**) Performance curve showing a SR-like bell-shaped with optimal improvement at an intermediate nGVS intensity. (**C**) Performance curve showing only optimal improvement at a particular nGVS intensity. (**D**) Performance curve showing none of the two.

**Figure 5:**
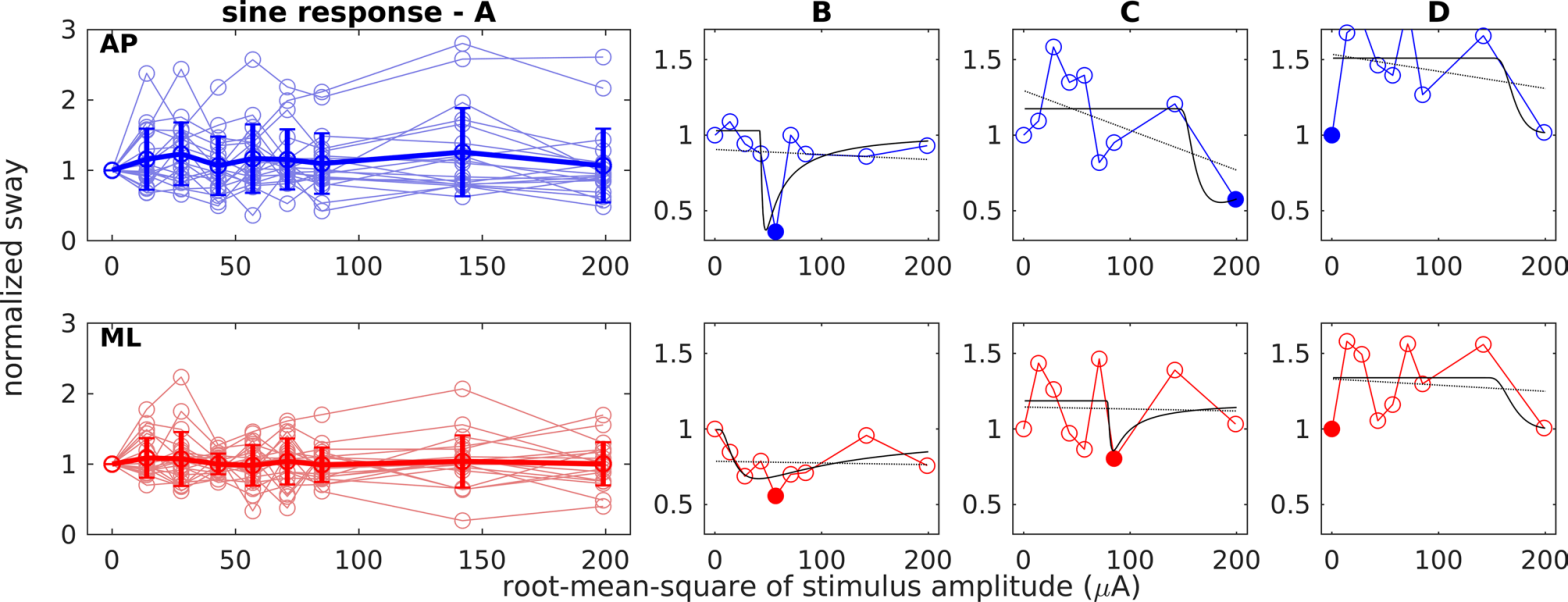
Normalized body sway response amplitudes for standing during sinusoidal platform tilts at varying nGVS intensities. Top row (AP, blue) shows anterior–posterior sway. Bottom row (ML, red) shows medio-lateral sway. (**A**) Responses of all subjects (light circles and lines) with group mean and standard deviation (thick line). (**B–D**) Three exemplary individual responses including the linear (grey) and the SR curve fit (black). Filled circles indicate minimum values. (**B**) Performance curve showing a SR-like bell-shaped with optimal improvement at an intermediate nGVS intensity. (**C**) Performance curve showing only optimal improvement at a particular nGVS intensity. (**D**) Performance curve showing none of the two.

In a second step of analysis, we examined whether response dynamics of body sway across the range of applied nGVS intensities might be actually compatible with the presence of vestibular-induced SR. SR-effects on body sway would be indicated by a pseudo-bell-shaped response curve with an optimal reduction of body sway at some particular nGVS intensities (Fig. 1D). For this purpose, sway parameters expressing a measure of overall body sway, i.e., sway path-length for quiet and sway-referenced stance conditions and sway amplitude in response to the tilt stimulus for sinusoidal tilt condition, were normalized with respect to the baseline condition (i.e., 0 µA nGVS) and plotted across the range of nGVS amplitudes. Figures 3, 4 and 5 depict group-average and representative individual body sway responses to nGVS and corresponding linear and SR curve fits for the three different examined stance conditions (individual plots for all recorded trials are provided as supplemental material).

In accordance with previous reports, body sway was reduced compared to baseline in at least one nGVS amplitude in the majority of subjects and platform conditions (quiet stance in AP direction 95% and ML 86%; sway-referencing AP 84% and ML 85%; sinusoidal tilt in AP 75% and ML 86%). In contrast, however, visual inspection of individual response dynamics by three human judges did only identify a minority of trials where body sway responses exhibited a SR-like pseudo-bell-shaped performance curve across applied nGVS intensities (quiet stance in AP direction 33% and ML 10%; sway-referencing AP 33% and ML 20%; sinusoidal tilt AP 25% and ML 24%). This observation was further supported by the finding that none of the SR-curve fits (both on an individual as well as on a group-average level) yielded a significantly better explanatory power as compared to the linear fits (this might, however, be partially explained by the relatively high number of degrees of freedom in the applied SR-curve model). In other words, body sway across tested nGVS amplitudes did not show any systematic changes, but rather showed random fluctuations.

## Discussion

Several previous studies reported that human postural sway can be reduced by the application of a particular intensity of nGVS^22–34^—an effect that was concordantly attributed to the exhibition of SR within vestibular signal transfer. However, none of these studies so far provided systematic evidence for the latter assumption. In this study, we therefore systematically examined whether SR-like changes in body sway across nGVS amplitudes can be actually observed in young healthy individuals. The exhibition of SR in a particular system is typically indicated by a noise-induced modulation of the system's output that follows a pseudo-bell-shaped performance curve with increasing noise intensity, which peaks at a particular optimal noise amplitude where the system's performance becomes optimally enhanced. In accordance with previous reports, we found reduced body sway (for all three tested balance conditions and AP as well as ML directions) at particular nGVS intensities in almost all participants. However, across the entire range of applied nGVS intensities, individual and grouped body sway modulations did not exhibit consistent performance curves, neither with respect to the curve fit nor the subjective, rater-based criterion. With respect to the latter subjective criterion, the absence of SR-like behavior was rather rated conservatively and some identifications of SR-like behavior might be disputed by other experienced raters. These ratings thus more or less provide an upper limit for the proportion of trials that can be considered to exhibit SR-like behavior. However, even based on this conservative rating strategy, only a minority of trials can be considered to exhibit SR-like responses. This observation suggests that, at least in young healthy individuals, reductions in body sway at particular nGVS amplitudes are likely to result from test–retest variations in the body sway parameters rather than being caused by vestibular SR.

Vestibular feedback cues play a minor role during quiet stance on a fixed surface, where postural adjustments predominantly rely on somatosensory and—to a lesser extent—visual cues^39,47^. We therefore focused on stance conditions that pronounce the role of vestibular feedback by (a) withdrawal of visual cues (eyes closure) and (b) by manipulating the proprioceptive reference to the Earth vertical through sinusoidal surface tilts or sway referencing. Even under these conditions we did not observe convincing evidence that nGVS induces SR-like reductions in body sway. Our observations are certainly limited by the focus on a young and healthy cohort, in which peripheral vestibular processing presumably operates at a near-to-optimal level and might thus leave little to no potential for externally induced improvements. It is thus conceivable, that SR-like changes in vestibular balance control might only be observable in the elderly^23^ or in patients with vestibular hypofunction^24,31^, where age-related or pathological vestibular hair cell degeneration has been associated to a decline in peripheral vestibular signal processing^48,49^.

However, SR-like effects using nGVS have been reported also for young healthy individuals. Galvan-Garza and colleagues observed in about 75% of young participants, that nGVS-induced modulations of the vestibular direction recognition threshold for passive motion perception were compatible with SR and followed a bell-shaped performance curve with optimal improvement at a particular intermediate nGVS intensity^18^. Analogously, it was demonstrated that nGVS effectively lowers the threshold to induce vestibulospinal reflex responses in about 90% of young participants^50^. Thus, the question arises why SR-like behavior can be found in perception and simple reflexes, but not in standing balance.

One reason could be differences between the processing of vestibular cues in ego motion, sensorimotor reflexes, and balance regulation. For instance, vestibulo-ocular reflex thresholds apparently differ from thresholds for vestibular motion perception, in particular in the low frequency range^51,52^, and exhibit different response dynamics to vestibular stimulation or visual-vestibular conflict^53,54^. Differences become even more apparent when comparing processing of vestibular cues at the perceptual and isolated reflex level to that during multisensory balance control. During standing with eyes closed, vestibular cues become integrated with proprioceptive cues and modulated by the feedback dynamics, the force generation process, and the multi-segmental body biomechanics of the standing subject^55^. Accordingly, vestibular input in postural control becomes considerably filtered and distorted^56^. In line with this, current models of postural control, assume that vestibular cues only become involved in balance regulation after multisensory fusion (in particular with proprioceptive cues) at a late processing stage that is close to the behavioral output^57,58^. Thus, comparisons between nGVS effects on different vestibular-related functions and output measures need to be considered carefully.

Another reason could be that psycho-physical estimates of vestibular perceptual thresholds, which showed SR-like effects evoked by nGVS^18^, are designed to yield excellent test-to-retest reliability^59,60^. In contrast, test-to-retest variations in standing balance are considerable^61,62^. Thus, inherent variations within the examined body sway measures might mask SR-like effects. However, earlier studies reported nGVS-induced reductions in body sway measures in the order of 10–40%^24,26^. The variability of sway measures across nGVS amplitudes from our current recordings had a standard deviation of 2% for quiet stance, 17% for sway-referencing trials, and 20% for sinusoidal platform tilt trials. Thus, at least the test-to-retest reliability during quiet stance would have been high enough to identify the previously reported 10–40% nGVS-induced sway reductions.

Finally, we observed that significant and consistent nGVS-induced body sway responses started to occur in ML direction for noise RMS intensities at and above 142 µA (approximately corresponding to nGVS peak amplitudes of 500–700 µA). In accordance in nonhuman primates, neuronal detection thresholds of primary vestibular afferents for GVS applied on the bilateral mastoid processes—a setup analogous to ours—were estimated to lie between peak amplitudes of 400–600 µA^63^. This suggests that vestibular SR in young healthy individuals with intact peripheral vestibular information processing, should be triggered, if any, by nGVS at amplitudes below these estimated peripheral thresholds. This assumption corresponds to the observation made by Galvan-Garza et al. that optimal nGVS-induced reduction in vestibular perceptual thresholds occurred at nGVS peak amplitudes at or below 500 µA^18^. In contrast, previous reports on nGVS-induced reductions of postural sway at stimulation amplitudes way above these estimated detection thresholds (e.g. 1000 µA^22,27,34^) are therefore unlikely to be attributable to vestibular SR. In these instances, reductions of body sway presumably rather result from a postural stiffening/stabilization response in the presence of an external induced vestibular disturbance as opposed to an effective facilitation of vestibular balance regulation.

## Supplementary Information

Supplementary Figure.

Supplementary Figure.

Supplementary Figure.

Supplementary Legends.

## Data Availability

The datasets generated and analyzed during the current study are available from the corresponding author on reasonable request.
