# Supplementary material for: No evidence for stochastic resonance effects on standing balance when applying noisy galvanic vestibular stimulation in young healthy adults
Source: Sci Rep. 2021 Jun 10;11:12327. doi: 10.1038/s41598-021-91808-w (PMC8192540; doi:10.1038/s41598-021-91808-w)
Supplement: Supplementary file 1 — Supplementary Figure. [file 41598_2021_91808_MOESM1_ESM.pdf]

Fixed surface condition (quiet stance)

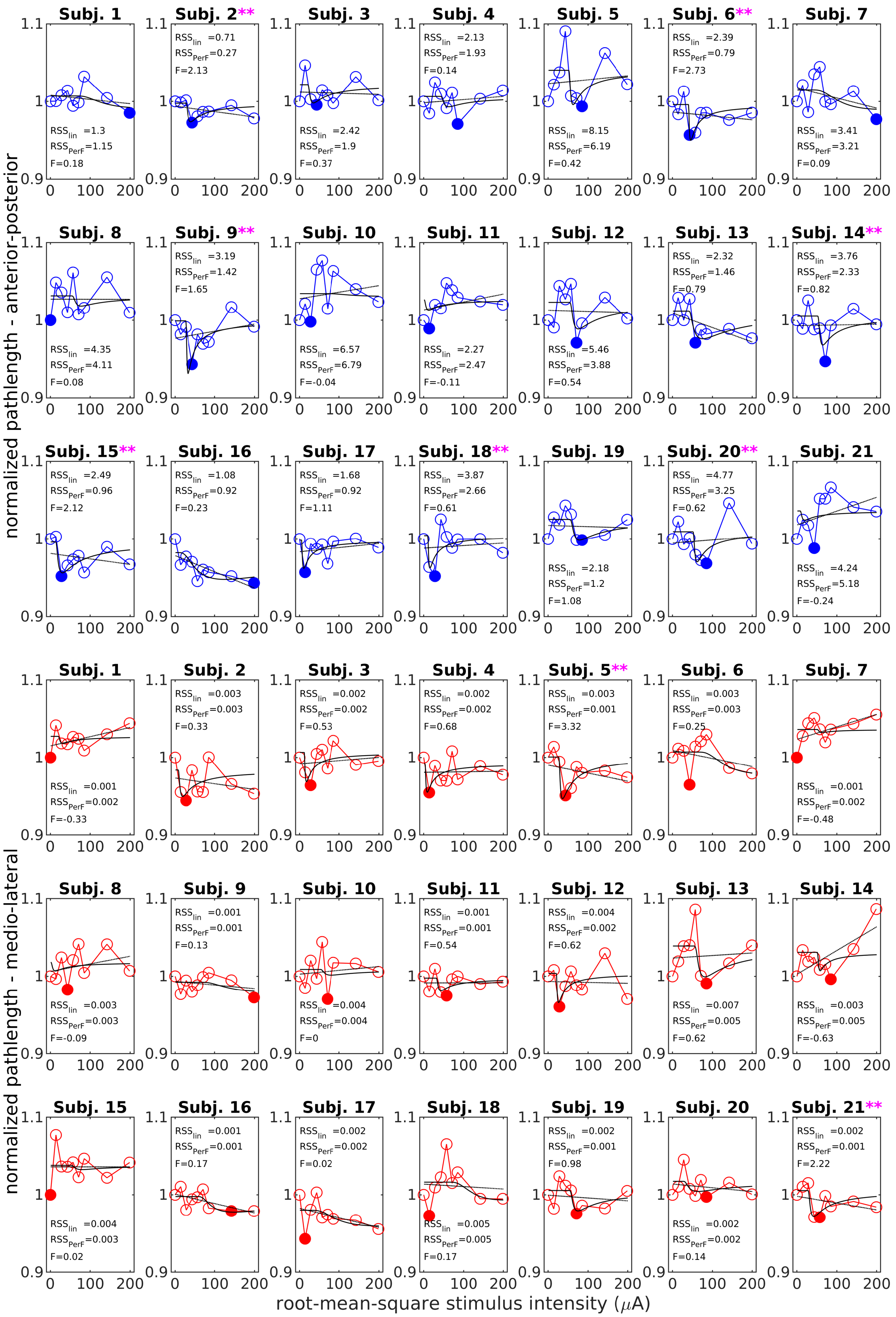

** indicate plots that were judged to follow bell-shaped curve by at least two of three judges

RSS values are given in units of 10^{-3}
