# Supplementary material for: No evidence for stochastic resonance effects on standing balance when applying noisy galvanic vestibular stimulation in young healthy adults
Source: Sci Rep. 2021 Jun 10;11:12327. doi: 10.1038/s41598-021-91808-w (PMC8192540; doi:10.1038/s41598-021-91808-w)
Supplement: Supplementary file 4 — Supplementary Legends. [file 41598_2021_91808_MOESM4_ESM.pdf]

No evidence for stochastic resonance effects on standing balance when applying noisy galvanic vestibular stimulation in young healthy adults

Assländer L[1], Giboin LS[1], Gruber M[1], Schniepp R[2,3], Wuehr M[2]

[1]Human Performance Research Centre, University of Konstanz, Konstanz, Germany [2]German Center for Vertigo and Balance Disorders (DSGZ), Ludwig-Maximilians-University, Munich, Germany, [3]Department of Neurology, Ludwig-Maximilians-University, Munich, Germany

Supplemental Figures

Legend: Figures show single subject data of the three support surface conditions ‚fixed surface‘, ‚sinusoidal tilt‘, and ‚sway referenced‘ for anterior-posterior (three top rows, blue) and medio-lateral (three bottom rows, red) sway direction. Note that for ‚fixed surface‘ both directions were recorded simultaneously. Each plot contains the linear fit and the fit to the periodic forcing function, where the residual sum of square values are given within each figure. F-values represent the statistical comparison between the two fits, taking the additional degrees of freedom of the periodic forcing function into account. Filled circles within each plot indicate the lowest value across nGVS stimulus intensity. Missing plots indicate that the subject was not able to perform the given condition (see manuscript for details).
